# Multi-host infection and phylogenetically diverse lineages shape the recombination and gene pool dynamics of *Staphylococcus aureus*

**DOI:** 10.1186/s12866-023-02985-9

**Published:** 2023-08-25

**Authors:** Stephanie S. R. Souza, Joshua T. Smith, Spencer A. Bruce, Robert Gibson, Isabella W. Martin, Cheryl P. Andam

**Affiliations:** 1 Department of Biological Sciences, University at Albany, State University of New York, Albany, NY USA; 2 Department of Molecular, Cellular and Biomedical Sciences, University of New Hampshire, Durham, NH USA; 3 Infectious Disease and Microbiome Program, Broad Institute of MIT and Harvard, Cambridge, MA USA; 4 New Hampshire Veterinary Diagnostic Laboratory, Durham, NH USA; 5 Dartmouth-Hitchcock Medical Center and Dartmouth College Geisel School of Medicine, Lebanon, NH USA

**Keywords:** *Staphylococcus aureus*, Population genomics, Host, Core genome, Accessory genome, Recombination

## Abstract

**Background:**

*Staphylococcus aureus* can infect and adapt to multiple host species. However, our understanding of the genetic and evolutionary drivers of its generalist lifestyle remains inadequate. This is particularly important when considering local populations of *S. aureus*, where close physical proximity between bacterial lineages and between host species may facilitate frequent and repeated interactions between them. Here, we aim to elucidate the genomic differences between human- and animal-derived *S. aureus* from 437 isolates sampled from disease cases in the northeast region of the United States.

**Results:**

Multi-locus sequence typing revealed the existence of 75 previously recognized sequence types (ST). Our population genomic analyses revealed heterogeneity in the accessory genome content of three dominant *S. aureus* lineages (ST5, ST8, ST30). Genes related to antimicrobial resistance, virulence, and plasmid types were differentially distributed among isolates according to host (human versus non-human) and among the three major STs. Across the entire population, we identified a total of 1,912 recombination events that occurred in 765 genes. The frequency and impact of homologous recombination were comparable between human- and animal-derived isolates. Low-frequency STs were major donors of recombined DNA, regardless of the identity of their host. The most frequently recombined genes (*clfB*, *aroA*, *sraP*) function in host infection and virulence, which were also frequently shared between the rare lineages.

**Conclusions:**

Taken together, these results show that frequent but variable patterns of recombination among co-circulating *S. aureus* lineages, including the low-frequency lineages, that traverse host barriers shape the structure of local gene pool and the reservoir of host-associated genetic variants. Our study provides important insights to the genetic and evolutionary factors that contribute to the ability of *S. aureus* to colonize and cause disease in multiple host species. Our study highlights the importance of continuous surveillance of *S. aureus* circulating in different ecological host niches and the need to systematically sample from them. These findings will inform development of effective measures to control *S. aureus* colonization, infection, and transmission across the One Health continuum.

**Supplementary Information:**

The online version contains supplementary material available at 10.1186/s12866-023-02985-9.

## Background

*Staphylococcus aureus* is a ubiquitous commensal and opportunistic pathogen of many vertebrate species. Often found on the skin and mucosal membranes of humans [1], it has been implicated in gastroenteritis, toxic shock syndrome, scalded skin syndrome, and invasive diseases such as bacteremia, pneumonia, endocarditis, and osteomyelitis [2]. In animals, it is known to cause mastitis in dairy-producing animals such as cattle and goats [3], ulcerative pododermatitis (or “bumblefoot”) in chickens [4], and skin and soft tissue infections in cats, dogs and horses [5]. The burden of *S. aureus* infections is exacerbated by the emergence and global spread of antimicrobial resistant strains [6]. Infections due to methicillin-resistant *S. aureus* (MRSA) have caused persistently high morbidity and mortality in humans worldwide [7–10]. Many MRSA strains also have the propensity to develop multi-drug resistance against other classes of antimicrobials beyond the beta-lactams [11, 12].

Recent population genomic studies of *S. aureus*, mostly focusing on populations spanning expansive geographical and temporal scales, have profoundly transformed current knowledge on *S. aureus* colonization, infection, and transmission. Host-switching has been a major feature of *S. aureus*’ evolutionary history. Domestication and global trade of animals that started during the agricultural revolution have accelerated host-switching events between humans, livestock, companion animals, and wildlife species [13, 14]. While certain *S. aureus* lineages appear to specialize in infecting one or few animal species (*e.g.*, sequence type (ST) 522 in small ruminants [15] and clonal complex (CC) 385 in avian species [16]), the acquisition or loss of host-adaptive genes is known to facilitate host-switching followed by subsequent adaptation [17, 18]. These host-adaptive genes are often located in mobile genetic elements such as phages, pathogenicity islands, and plasmids [19–21]. After switching to a new ecological host niche, further host-specific mutations permit its expansion into new host populations [22]. The ability to colonize multiple host species and to readily take up DNA [13, 23] means that reservoirs of novel resistance mechanisms and genetic variants will persist in the environment. Hence, MRSA lineages that are able to cross between hosts from healthcare, community, and veterinary settings can rapidly disseminate and pose a significant risk to the human population.

Nonetheless, our understanding of the genetic and evolutionary drivers of the generalist lifestyle of *S. aureus* remains inadequate. In local populations of *S. aureus*, close physical proximity between bacterial lineages and between host species may facilitate frequent and repeated interactions between them. Co-circulation of bacterial lineages may consequently lead to pervasive sharing of genetic material within the population, which can foster genetic homogeneity [24]. We hypothesize that infection of different hosts represents a strong selective pressure that can influence the structure and recombination dynamics of the local gene pool of co-circulating *S. aureus*. Here, we analyzed 437 genomes to elucidate the gene content differences between human- and animal-derived *S. aureus* isolates sampled from disease cases in northeast United States. We discuss the importance of understanding the genetic basis of the host generalist lifestyle in *S. aureus* to inform future efforts for targeted mitigation of public health threats, especially in local populations where co-circulation and interaction of lineages and hosts are more prevalent.

## Results

### Phylogenetic relationships and population structure

**Fig. 1:**
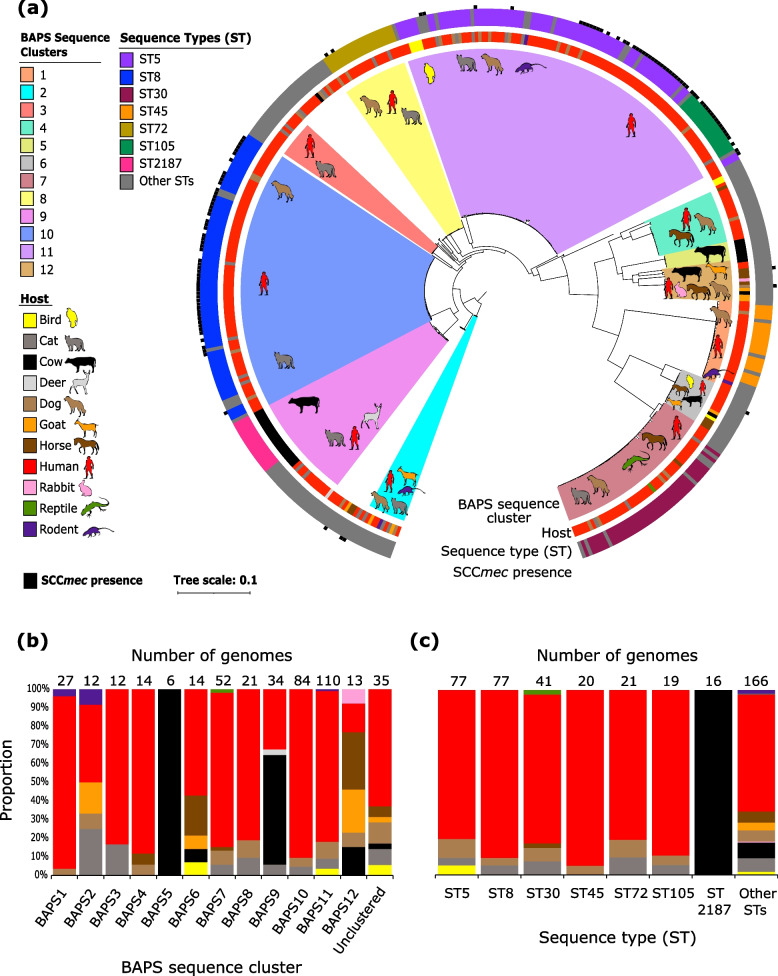
Phylogenetic relationships and population structure of 437 *S. aureus* isolates from animal and human hosts. **a** Maximum likelihood tree showing the phylogenetic relationships of 437 *S. aureus* genomes. The tree was built using the SNPs extracted from 1,586 core genes. The tree was rooted using the BAPS Cluster 2, which was closest to the outgroup (*Staphylococcus argenteus* MSHR1132 (NCBI accession number: GCA_000236925.1; Supplementary Fig. 1). Colored branches represent the 12 sequence clusters inferred by the program BAPS. Within each cluster, the hosts from which isolates in the cluster were derived from are shown in the shaded branches. The three outer rings represent the host, sequence type (ST), and the presence of the SCC*mec* in each genome. Branch scale represents the number of nucleotide substitutions per site. **b** Proportion and distribution of *S. aureus* isolates by host within each sequence cluster. **c** Proportion and distribution of *S. aureus* isolates by host within each major ST. For panels b and c, the total number of genomes in each category are shown on top of the bars and the colors of the bars correspond to the color legend of hosts in panel a

We obtained 437 high-quality draft genomes of *S. aureus* that we have previously published (Supplementary Table S1). These came from pure cultures of single colonies derived from clinical specimens. Of these, 323 isolates were obtained from the blood of unique pediatric and adult patients with bacteremia at the Dartmouth-Hitchcock Medical Center (DHMC), New Hampshire, United States, from 2010 to 2018 [25]. The remaining 114 isolates were derived from clinical specimens of ten animal species that were diagnosed with disease from 2017 to 2020 at the New Hampshire Veterinary Diagnostic Laboratory (NHVDL) [26]. To examine the phylogenetic relationships of animal- and human-derived isolates, we constructed a maximum likelihood tree based on 97,912 single nucleotide polymorphisms (SNP) extracted from 1,586 core genes present in ≥ 99% of the genomes in our dataset (Fig. 1a and Supplementary Figure S1).

Multi-locus sequence typing (MLST) revealed the existence of 75 previously recognized sequence types (ST), but many of these are low-frequency STs. The most prevalent STs were ST5 (77/437 genomes representing 17.6% of the population), ST8 (77/437 genomes or 17.6%), and ST30 (41/437 genomes or 9.4%). These three dominant STs (ST5, ST8, ST30) consisted of isolates from multiple hosts, which were mainly humans, dogs, and cats (Fig. 1a). Five isolates have major mismatches with one of the genes in the MLST scheme of *S. aureus* [27].

Using Bayesian analysis of population structure (BAPS) hierarchical clustering of the core genome alignment, 12 distinct monophyletic sequence clusters can be delineated in the population. A total of 35 genomes could not be classified within any of the 12 clusters and represented low-frequency genotypes scattered throughout the phylogeny. The largest sequence cluster BAPS11 contained 110 genomes representing 12 STs (77 genomes from ST5, 19 genomes from ST105, and 14 genomes from ten other STs). Another large cluster is BAPS10, which consisted of 84 genomes representing six STs (77 genomes from ST8, two genomes from ST1181, two genomes from ST1750, and one genome each from ST2253, ST6400, ST6418). Together, these two sequence clusters represented 44.4% (194/437 genomes) of the *S. aureus* population, of which 90.5% (76/84 genomes) in BAPS10 and 80.9% (89/110 genomes) in BAPS11 comprised human isolates (Fig. 1b). The third largest sequence cluster BAPS7 consisted of 41 genomes from ST30, four genomes from ST34, and the remaining genomes from seven other STs.

Other STs in the population were ST72 (representing BAPS8; 21 genomes), ST2187 (BAPS9; 16 genomes) and ST45 (BAPS1; 20 genomes) (Fig. 1c). The remaining low-frequency STs (68 STs) comprised 166 genomes, with some of these STs representing variants of known STs. The most diverse sequence cluster was BAPS12, which consisted of isolates representing five STs sampled from six animal species (cow = 2, dog = 1, goat = 3, horse = 4, human = 2, rabbit = 1). In contrast, sequence cluster BAPS5 consisted of isolates from ST151 and all were derived from cows.

### Genome content varies between human- and animal-derived isolates

**Fig. 2:**
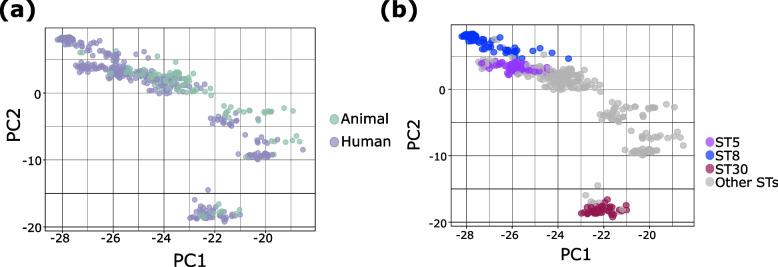
Principal component analysis (PCA) of the *S. aureus* accessory genome. PCA based on the presence or absence of accessory genes, with dots representing isolates colored according to host (**a**) and according to ST (**b**). For visual clarity, only the three dominant STs (ST5, ST8, ST30) are colored. PC1 and PC2 are principal component 1 and principal component 2, respectively

We examined the overall genome-wide variation, if any, within the *S. aureus* population. We used principal component analysis (PCA) to illustrate the maximum variance based on the presence or absence of genes in the accessory genomes. Results show that isolates from human and animal hosts have overlapping accessory genome content (Fig. 2a). When we partitioned the PCA plots based on STs, results reveal that the accessory genomes of each ST were distinct from each other (Fig. 2b). The accessory genomes of ST30 were highly divergent from ST5 and ST8 genomes, which mirrors their phylogenetic relationships based on the core genome (Fig. 1a). While ST5 and ST8 genomes were more similar, we can still observe slight differences between them. These results showed that differences in the *S. aureus* infection of human versus animal hosts of the three dominant STs is partly attributed to lineage-associated variation in accessory genomes.

We next sought to precisely determine which genetic components varied between human- and animal-derived *S. aureus*. When partitioned according to host, human-derived isolates have a mean genome size of 2.79 Mb (range: 2.66—2.97 Mb), which was slightly higher than that of the animal-derived isolates (mean = 2.77 Mb; range: 2.68—2.90 Mb) (*p* = 4.55e-06; Welch’s t-test) (Supplementary Figure S2a). The largest genome belonged to an ST8/BAPS10 isolate, while the smallest genome was an ST59/BAPS4 isolate. Both isolates were obtained from human specimens. Human-derived isolates also have significantly higher number of coding sequences (CDS) per genome (Supplementary Figure S2b). Human-derived isolates have 2,616 CDS per genome on average (range: 2,440 – 2,814 CDS), while animal-derived isolates have 2,570 CDS per genome on average (range: 2,429 – 2,739) (*p* = 2.47e-09; Welch’s t-test).

We then characterized the variation in genes that were present in ≥ 99% of the genomes (*i.e.*, 433 out of 437 genomes), which we defined as the core genome. Using the core genome alignment, we calculated the genetic distance between every pair of genomes in each group (Supplementary Figure S2c). The overall core SNPs distance was 12,043 SNPs. The number of core SNPs in animal isolates ranged from 0 to 24,152 SNPs (mean = 12,038 SNPs), whereas it was 0 to 24,171 SNPs (mean = 12,044 SNPs) in human isolates. We did not find any significant difference in the mean core genetic distance between animal-derived isolates and human-derived isolates.

We considered all other genes that were detected in < 99% of the genomes as part of the accessory genome. We found that human-derived isolates also have a significantly higher number of accessory genes per genome than animal-derived isolates (Supplementary Figure S2d). Human-derived isolates have on average 1,010 accessory genes per genome (range: 835 – 1,202 genes), while animal-derived isolates have on average 983 accessory genes per genome (range: 841 – 1,141 genes) (*p* = 3.57e-04; Welch’s t-test). However, animal-derived isolates were significantly enriched in genes that are isolate-specific (or unique genes) when compared to human-derived isolates (Supplementary Figure S2e). The mean number of unique genes in animal isolates was 9.73 genes per genome (range: 0 – 117), it was 4.63 genes per genome (range: 0 – 51) in human isolates (*p* = 1.41e-03; Welch’s t-test). A total of 18.3% (80/437) of the genomes did not carry unique genes, of which 80% (64/80) were from human hosts (Supplementary Table S1). The genome with the highest number of unique genes was retrieved from a cow and belonged to ST1245/BAPS12.

Of the 2,607 unique genes extracted from both human and animal isolates groups, we were able to assign 1,762 genes (equivalent to 67.6%) to 20 functional categories based on the eggNOG orthology prediction and functional annotation (Supplementary Table S2). These 20 categories can be sorted into three broad functional groups: cellular processes and signaling, information storage and processing, and metabolism. From the 1,497 unique genes extracted from the genomes of human-derived isolates, 69.5% (1,041/1,497) were classified in at least one functional category. In animal isolates, the 65.5% (*n* = 721 genes) of the 1,110 unique genes could be assigned to a functional category. For those unique genes with inferred functions, three functional categories were highly represented: (1) replication, recombination and repair, (2) metabolism and transport of amino acids, (3) cell wall/membrane/envelope biogenesis (Supplementary Table S2). Although unique genes in these three categories were abundant in both human- and animal-derived isolates, they were slightly more frequent in human isolates. A total of 4.7% (49/1,041) of the human and 3.7% (27/721) of the animal unique genes were assigned in more than one functional category. Unique genes classified as having unknown function were predominant in both groups.

### Host-associated differences in antimicrobial resistance (AMR), virulence, and plasmids

**Fig. 3:**
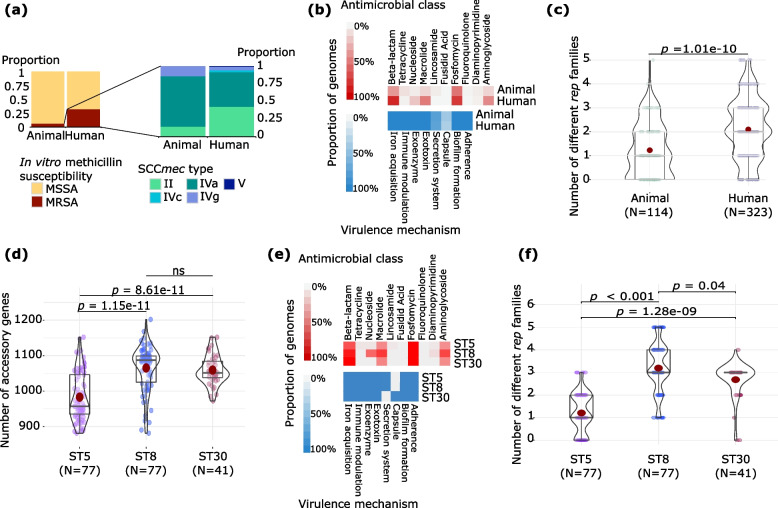
Composition of the *S. aureus* accessory genomes. **a-c** Comparison of human- and animal-derived isolates showing the (**a**) distribution of SCC*mec* elements, (**b**) distribution of AMR and virulence determinants, and (**c**) the number of different *rep* families. **d-f** Comparison of the three dominant STs (ST5, ST8, and ST30) showing the (**d**) the number of accessory genes, (**e**) distribution of AMR and virulence determinants, and (**f**) the number of different *rep* families. Intensity of box shading in panels b and e indicates the proportion of genomes harboring at least one gene conferring resistance to an antimicrobial class (red) and virulence mechanism (blue). In panels c, d and f, colored dots represent genomes in each category. In the violin plots, the mean value is represented by the red dot, box represents the interquartile range, horizontal line in the middle of the box represents the median, and the lower and upper whiskers represent the lowest data point without the outliers and the highest data point without outliers, respectively. Details of the distribution of specific genes related to AMR, virulence and plasmids are presented in Supplementary Table S1

We next examined host-associated differences in *S. aureus* in terms of the accessory genetic elements associated with AMR and virulence. First, we characterized the distribution of the mobile element staphylococcal chromosomal cassette *mec* (SCC*mec*) that carries the determinant for beta-lactam resistance encoded by the *mecA* gene [28]. SCC*mec* was detected in different STs as seen in the core genome phylogeny (Fig. 1a). When sorted by their hosts, SCC*mec*-carrying genomes represented 32.2% (104/323 genomes) of the human-derived *S. aureus*, whereas SCC*mec*-carrying genomes represented 6.1% (7/114 genomes) of the animal-derived isolates (Fig. 3a). Of the 14 known structurally distinct types of SCC*mec* [28–30], we detected five that were present in our dataset. Of these, the most common were types IVa and II, which were present in 56 and 45 of the 111 SCC*mec*-carrying genomes, respectively. When classified according to host, we detected a total of five SCC*mec* types (II, IVa, IVc, IVg, V) among the human-derived genomes, whereas only three types (II, IVa, IVg) were present in animal-derived genomes.

Using in silico identification of AMR genes, we detected a total of 26 genes that were associated with resistance to ten antimicrobial classes (aminoglycoside, diaminopyrimidine, fosfomycin, fusidic acid, lincosamide, macrolide, nucleoside, tetracycline, and beta-lactam) (Fig. 3b and Supplementary Table S1). Across the entire *S. aureus* population, we detected a mean of three AMR genes per genome (range: 0 – 12). The isolate carrying the highest number of AMR genes in its genome (*n* = 12 AMR genes) came from a human isolate and belonged to ST5/BAPS11. When classified according to host, animal-derived isolates harbored on average 1.5 AMR genes per genome (range: 0 – 10), whereas human-derived isolates carried 3.5 AMR genes per genome (range: 0 – 12). Among those isolates from animals, an ST5/BAPS11 isolate recovered from a dog host harbored the highest number of AMR genes (*n* = 10 AMR genes). In terms of specific AMR class, we found that human-isolates isolates were enriched in genes associated with resistance to aminoglycosides, beta-lactams, fosfomycin, and macrolides compared to animal-derived isolates. Among the human isolates, we detected 43.6% (or 14/323 genomes), 85.1% (275/323 genomes), 76.8% (or 248/323 genomes), and 50.2% (162/323 genomes) that carry at least one gene conferring resistance to aminoglycoside, beta-lactam, fosfomycin, and macrolide, respectively. In contrast, we detected 14.0%% (or 16/114 genomes), 37.7% (43/114 genomes), 52.6% (or 60/114 genomes), and 13.1% (15/114 genomes) of animal isolates carrying at least one gene conferring resistance to aminoglycoside, beta-lactam, fosfomycin, and macrolide, respectively. The genes *qacA* (fluoroquinolone), *fusC* (fusidic acid), *dfrC* and *dfrG* (diaminopyrimidine), *lnuA* and *lnuG* (lincosamide) were rarely detected in both groups of isolates, all of which were detected in less than 5% of the genomes.

We also compared the presence of virulence genes between human- and animal-derived isolates (Fig. 3b and Supplementary Table S1). We identified 47 distinct genes and 50 other genes that are part of seven different operons that were associated with eight classes of virulence mechanisms. For clarity, we counted each operon as a single unit if all genes in that operon were present when identifying virulence determinants. The virulence determinants that were most commonly detected were associated with exotoxin production. Across the entire population, we identified a total of 21 distinct genes and three operons associated with exotoxin production. On average, human- and animal-derived isolates carried 36 and 32 virulence determinants, respectively. Both human- and animal-derived isolates were also enriched in genes related to iron acquisition, immune modulation, exoenzyme, biofilm formation, and adherence. All genomes in both groups carried at least one gene related to these six classes of virulence mechanisms (Supplementary Table S1). Among the human-derived isolates, we detected the presence of the operons related to capsule formation (Cap) and Ess/Type VII secretion system in 35% (113/323 genomes) and 74.3% (240/323 genomes) of the genomes, respectively. Among animal-derived isolates, these two operons were present in 41.2% (47/114 genomes) and 77% (88/114 genomes), respectively.

Lastly, we also compared the number of plasmids between human- and animal-derived isolates. We used the presence of the gene that encodes the plasmid-encoded Rep initiator protein as an indication of the presence of a plasmid [31]. Across the entire *S. aureus* population, we identified a total of 62 distinct *rep* genes from five *rep* families (Fig. 3c and Supplementary Table S1). The most diverse and frequently found *rep* family was the repA_N, which consisted of 20 different *rep* genes present in 216 genomes. This approach did not allow us to precisely determine the number of plasmids present in each genome; instead, we focused on comparing the number of *rep* families between human- and animal-derived isolates (Fig. 3c). *S. aureus* from human hosts carried a mean of two *rep* families (range: 0 – 5), whereas *S. aureus* from animal hosts harbored a mean of one *rep* family (range: 0 – 5) (*p* = 1.01e-10; Welch’s t-test). We did not detect any *rep* genes in 18% (79/437) of the genomes. We found a total of nine genomes that carry all five *rep* families, of which one came from a cat (ST1) and eight from humans which were all members of sequence cluster BAPS10 (seven from ST8 and one from ST1750).

### Genome content varies among the three dominant STs

Three STs (ST5, ST8, ST30) dominate our *S. aureus* population, all of which were detected in both human and animal hosts. We therefore sought to determine the gene content differences among them. First, we calculated the genome-wide average nucleotide identity (ANI) values for every pair of genomes that belonged to each ST (Supplementary Figure S3 and Table S3). ANI values between ST5 and ST8 genomes ranged from 98.60% to 99.06% (mean = 98.90%), 97.41% to 97.87% (mean = 97.64%) between ST5 and ST30 genomes, and 97.36% to 97.73% (mean = 97.58%) between ST8 and ST30 genomes. ST8 and ST30 genomes had significantly more accessory genes than ST5 genomes (both comparisons with *p* < 0.05) (Fig. 3d). ST8 and ST30 carried on average 1,065 and 1,059 accessory genes per genome, respectively, while ST5 genomes carried 982 accessory genes per genome.

We found differences in the distribution of AMR genes among the three STs (Fig. 3e). Genomes that comprised ST8 were enriched for genes related to nucleoside resistance. The gene *sat-4*, which encodes a streptothricin acetyltransferase conferring resistance to the streptothricin (a nucleoside antibiotic) [32], was detected in 57% or 44/77 genomes that make up ST8. In contrast, only 3.9% or 3/77 genomes of the ST5 isolates carried this gene, whereas it was not detected in ST30 isolates. ST8 genomes were also enriched for genes related to macrolide and aminoglycoside resistance. The proportion of ST8 genomes carrying at least one macrolide resistance gene and one aminoglycoside resistance gene was 76.6% (59/77 genomes) and 71.4% (55/77 genomes), respectively. In contrast, the same genes were present only in 40–43% of the ST5 and ST30 genomes. We also found ST-associated differences in genes related to aminoglycoside and macrolide resistance. ST8 genomes were enriched for the genes *aph(3’)-IIIa* (aminoglycoside), *mphC,* and *msrA* (macrolide), which we detected in 66.2% (51/77 genomes), 71.4% (55/77 genomes), and 70.1% (54/77 genomes), respectively. We also found that the SCC*mec* element was present only in genomes from ST5 and ST8, but not in ST30 (Fig. 1a and Supplementary Table S1). The frequency and diversity of this element varied between ST5 and ST8. A total of 39% (30/77) of the ST5 genomes carried SCC*mec*, predominantly type II (76.7% – 23/30 genomes). In contrast, 66.2% (51/77 genomes) of ST8 genomes carried SCC*mec* and were mostly type IVa (92.1%—47/51 genomes) (Supplementary Table S1).

We also compared the virulence genes present in the three STs (Fig. 3e). The three STs were all enriched in genes related to iron acquisition, immune modulation, exoenzyme production, exotoxin production, biofilm formation, and adherence. However, we observed differences in the distribution of genes related to capsule biosynthesis and secretion system among the three STs. We did not detect the capsule genes *capH*, *capI*, *capJ,* and *capK* in any genome belonging to ST5 and ST8 (Supplementary Table S1). As we mentioned before, we only considered complete operons if all the genes that make up that operon were present. In ST30, only two genomes did not carry the complete capsule operon due to the absence of the *capP* and *capI* genes. In terms of the Ess/Type VII secretion system, ST5 and ST8 genomes were both enriched for genes related to this operon. Only one genome from ST5 (isolate from a dog) did not carry the complete functional structure of the *ess* operon, as a result of a missing *essC* gene. The *essC* gene was completely absent in all ST30 genomes.

We also detected differences in the diversity of *rep* families in these three STs (Fig. 3f). ST8 genomes carried three *rep* families per genome (range: 1 – 5), whereas ST5 and ST30 genomes carried one (range: 0 – 3) and two (range: 0 – 4) *rep* families per genome, respectively (all comparisons were *p* < 0.05). The *rep* family Rep_trans was present in all ST8 genomes, while the *rep* families Inc18 (79.2%—61/77 genomes) and RepA_N (76.6% or 59/77 genomes) were also detected. In ST5 genomes, the RepA_N family was predominant (62.3% or 48/77 genomes). In ST30, the *rep* families Inc18 and rep3 were both present in 92.7% or 38/41 of the genomes.

Finally, we also sought to explore the differences between human- and animal-derived isolates within each of the three major STs. We detected significant differences in the number of accessory genes per genome between human- and animal-derived isolates in ST8, but not in ST5 and ST30 (Supplementary Figure S4a). The number of different *rep* plasmid families per genome also varied significantly between human- and animal-derived isolates in ST5 and ST8, but not in ST30 (Supplementary Figure S4b). In terms of the presence of AMR determinants, all genomes regardless of whether they were derived from human or animal and regardless of their ST affiliation carried genes associated with resistance to fosfomycin. However, human- and animal-derived isolates within the three STs differed in the prevalence of genes associated with resistance to aminoglycoside, macrolide, nucleoside, tetracycline, and beta-lactam (Supplementary Figure S4c). The human- and animal-derived genomes within the three STs carry similar sets of virulence determinants (Supplementary Figure S4c). We also noted that human- and animal-derived genomes in ST5 and ST8 lacked genes related to capsule formation (Cap), while human- and animal-derived genomes in ST30 lacked genes related to the Ess/Type VII secretion system.

Overall, our results showed that accessory gene content, including AMR genes, virulence determinants and plasmids, varied among isolates from different phylogenetic lineages (STs) as well as among isolates from different hosts. We also observed some gene content differences in isolates belonging to the same ST (ST5 and ST8) but sampled from different hosts.

### Frequent recombination in the core genome

**Fig. 4:**
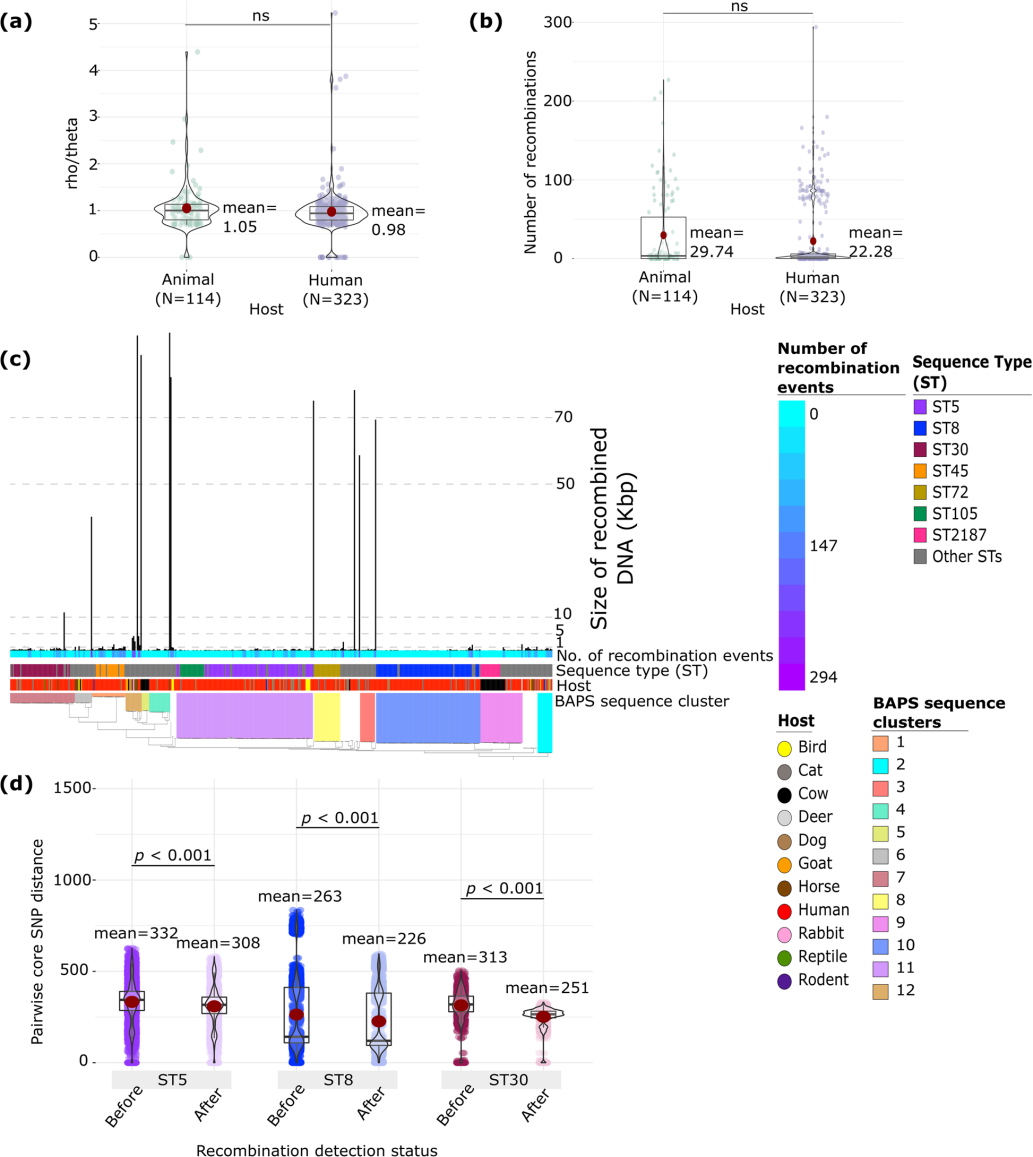
Homologous recombination in human- and animal-derived *S. aureus* isolates detected by ClonalFrameML. **a** Comparison of the ρ/θ ratios between human and animal isolates. **b** Comparison of the number of recombination events detected in human and animal isolates. In panels a and b, colored dots represent genomes in each category. **c** Total length of recombined DNA per genome and the number of recombination events detected (box shading) per genome. The tree is identical to that in Fig. 1a. The list of all recombination events are presented in Supplementary Table S4. **d** Comparison of the pairwise core genome SNP distance before and after recombined DNA was removed. In panel d, each dot represents comparison between a pair of genomes. In the violin plots, the mean value is represented by the red dot, box represents the interquartile range, horizontal line in the middle of the box represents the median, and the lower and upper whiskers represent the lowest data point without the outliers and the highest data point without outliers, respectively

We next sought to compare the frequency of homologous recombination in the genomes of human- and animal-derived isolates. Out of the 437 isolates, 320 (or 73.22%) had at least one recombination event detected in the core genome (Supplementary Table S4). First, we estimated the relative rates at which polymorphisms were introduced in the core genes via recombination (rho ρ) and mutation (theta θ). For those isolates that experienced recombination, we estimated this ρ/θ ratio to be 1.11. We did not find significant difference in the ρ/θ ratios when we partitioned the genomes according to whether they came from human or animal hosts (Fig. 4a). We also inferred the number of recombination events that occurred in the core genome. Among those isolates that experienced recombination, we estimated an average of 33 recombination events per genome (range = 1 – 294). We did not detect significant difference in the number of recombination events when we partitioned the genomes according to host (Fig. 4b). Animal-derived isolates experienced on average 29 recombination events, whereas human-derived isolates experienced on average 22 recombination events.

For every isolate that experienced recombination, we also calculated the total length of recombined DNA segments in the core genome (Fig. 4c). Overall, the total number of base pairs per genome that had been impacted by recombination was 2,467 bp (mean). When partitioned according to host, recombination affected a total length of 4,631 bp (mean) in animal-derived isolates and 1,746 bp (mean) in human-derived isolates (*p* = 0.19; Welch’s t-test). There were eight genomes that contained more than 50,000 bp of core genome that experienced homologous recombination. Among these eight hyper-recombinant genomes, four were from humans (from ST12, ST20, ST22, ST88), one from dog (from ST1290), one from cow (BAPS12/ST1245), and two from horses (BAPS4/ST1640 and BAPS12/ST816).

Next, we compared the impact of homologous recombination in the three dominant STs (ST5, ST8, ST30). For each ST, we compared the genetic distance calculated from the SNPs of the core gene alignment for every pair of genomes before and after recombined DNA segments were removed (Fig. 4d). Results from all three STs revealed that recombination greatly contributed to the genetic variation of the ST-specific core genomes (*p* values < 0.001 for each of the three STs; Welch’s t-test). Comparing the three STs, we found that ST5 genomes differed by 332 core SNPs on average (range: 0 – 627 SNPs) for all possible pairs of genomes. This was significantly greater than the 313 SNPs (range: 0 – 504 SNPs) among ST30 genome pairs (*p* = 1.49e-11; Welch’s t-test) and 263 SNPs (range: 0 – 835) among ST8 genome pairs (*p* < 0.001; Welch’s t-test). Comparing ST8 and ST30, we found that the core genome of ST30 was more impacted by homologous recombination than ST8 (*p* = 3.70e-12; Welch’s t-test).

### Genome-wide recombination across STs and hosts

**Fig. 5:**
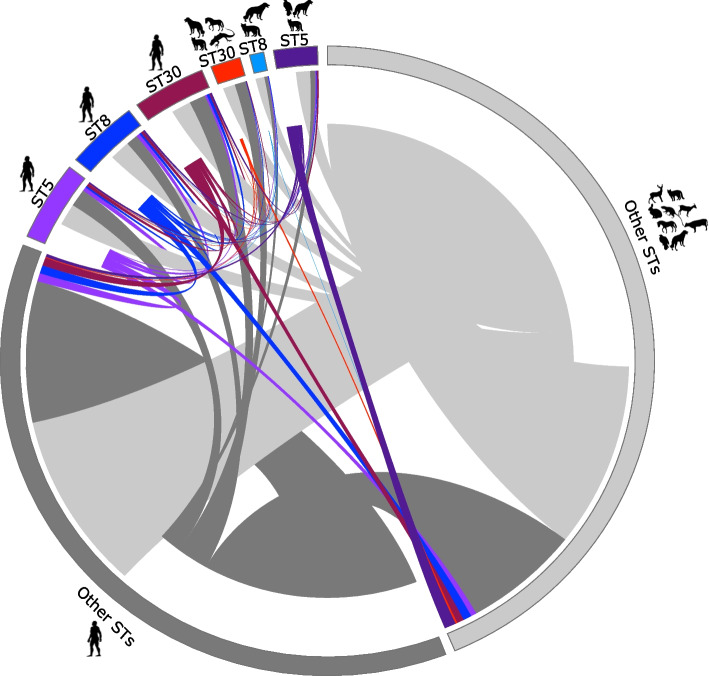
Donors and recipients in recombination events of core and shared accessory genes inferred by fastGEAR. For each of the three dominant STs (ST5, ST8, ST30), we subdivided the genomes into whether they came from a human host or animal host. We grouped all low-frequency STs into the Other STs category, which were further subdivided into whether they were human-derived (dark gray outer ring) or animal-derived (light gray outer ring). The length of the outer block is proportional to the number of genomes in which recombination was detected. Each line in the center connects a pair of donor and recipient genomes. For each line, the recipient genome is indicated by the end of the line ending nearer the outside of the plot and the potential donor cluster is indicated by the end of the line nearer the center of the plot. The color of the connecting line is based on the color of the recipient genome. The width of the lines in the center is proportional to the number of recombination events between a pair of genomes from the respective groups. The list of all recombination events is presented in Supplementary Table S5

We also determined recombination events in core and shared accessory genomes, and for every recombination event, we also identified the donor and recipient genomes (Fig. 5 and Supplementary Table S5). Across the entire *S. aureus* population, we identified a total of 1,912 recombination events that occurred in 765 genes. Figure 5 shows the donor-recipient recombination partners within and between STs. For visual clarity, we grouped all low-frequency STs into the Other STs category, which was further subdivided into whether they were human-derived (dark gray outer ring) or animal-derived (light gray outer ring). We found that the most frequent recombination partners were isolates from the Other STs category from both human and animal hosts. We detected a total of 407 recombination events that involved animal isolates from the Other STs category that acted both as donor and as recipient of recombined DNA. We identified 248 recombination events involving genomes from the Other STs category whereby the donors were human-derived isolates, and the recipients were animal-derived isolates. In contrast, we identified 391 recombination events involving genomes from the Other STs category whereby the donors were animal-derived isolates, and the recipients were human-derived isolates. When a subsample of the dataset was reanalyzed using the same number of human and animal isolates (114 genomes for each host group), we identified a total of 1,477 recombination events in 585 genes (Supplementary Table S5). In this second (subsampled) dataset, we also found that the Other STs category from both human and animal hosts were the most frequent partners, confirming the results found for the entire dataset (Supplementary Figure S5 and Supplementary Table S6).

The three dominant STs (ST5, ST8, ST30) were also frequently involved in recombination. We first identified the recombination events within each ST regardless of the identity of the host. In ST5, we detected a total of 248 recombination events, of which 92 involved an ST5 donor and 156 involved an ST5 recipient. In ST8, we detected a total of 171 recombination events, of which 53 involved an ST8 donor and 118 involved an ST8 recipient. In ST30, we detected a total of 203 recombination events, of which 59 involved an ST30 donor and 144 involved an ST30 recipient. However, recombination between a human-derived and an animal-derived isolate of the same ST was not frequently observed. For example, only five of the total 248 recombination events (2.01%) detected in ST5 occurred between genomes from different hosts. In ST8, only one of the total 171 recombination events (0.58%) detected occurred between genomes from different hosts. In ST30, only five of the total 203 recombination events (2.5%) detected occurred between genomes from different hosts.

In summary, these results showed that recombination has impacted the *S. aureus* population regardless of the host identity of the isolates. All three major STs were also frequent recipients of recombined DNA from members of the Other STs category from both animal and human hosts.

### Three genes associated with host infection and virulence are frequently recombined

Across the entire *S. aureus* population, the most frequently recombined genes were *clfB*, *aroA*, and *sraP* (Supplementary Table S5). The gene *aroA* was detected in all genomes and hence is part of the core genome. The genes *clfB* and *sraP* were detected in 326 and 385 genomes, respectively, and are thus part of the accessory genome.

The *clfB* gene encodes the fibrinogen-binding clumping factor B protein in *S. aureus* [33]. Among the 24 recombination events involving this gene, 17 (70.8%) involved a human-derived isolate acting as a DNA donor and which came from the low-frequency STs (*i.e.*, Other STs category). Most of the recipients of the *clfB* recombination were also human-derived isolates from genomes in the Other STs category (14/24 or 58.3%). This gene was also recombined in human-derived ST30 isolates, with two recombination events involving human-derived ST30 as the donor and one involving human-derived ST30 as the recipient. Another frequently recombining gene was *aroA*, which functions in the biosynthesis and transport of aromatic compounds [34] and has been implicated in biofilm formation and virulence [35]. The donor lineages of all recombination events involving *aroA* were animal-derived isolates from the Other STs category. Among the 20 recombination events of *aroA*, eight (40%) involved animal-derived isolates from the Other STs category as recipient lineages. Human-derived isolates from Other STs category were also recipients in six *aroA* recombination events (30%). Lastly, we also detected frequent recombination in the gene *sraP*, which functions in human platelet binding [36]. The gene *sraP* was implicated in 20 recombination events, of which 16 involved donor lineages from human-derived isolates from the Other STs category. We also identified four *sraP* recombination events whereby animal-derived isolates from the Other STs category acted as donors. The recipient lineages of all recombined *sraP* were all from the Other STs category, regardless of host identity (10 events each). We further examined the phylogenetic relationships of *S. aureus* in terms of these three recombining genes (Supplementary Figure S6). Although isolates from each of the three dominant STs mainly grouped together, the topologies of the three gene trees showed incongruencies among them and when compared to the core genome tree. Many less common STs were interspersed within each of the three dominant STs, further suggesting the presence of recombination between them.

## Discussion

Understanding the genetic and evolutionary factors that contribute to the generalist and host-switching lifestyle of *S. aureus* is critical to developing measures to control colonization, infection, and transmission. Here, we used population genomic approaches to compare human- and animal-derived *S. aureus* from disease cases in the northern New England region located in the northeast United States. Our results show that genomic variation in *S. aureus* is partly attributed to the differences between lineages as well as differences in their host distribution. Frequent but variable patterns of recombination among *S. aureus* lineages, including the low-frequency lineages, and traversing host barriers further shape the local gene pool structure and reservoir of host-associated genetic variants.

The co-circulation of *S. aureus* lineages within a defined geographical range and time period can lead to widespread sharing of genetic material between them, which may facilitate increased genetic homogeneity in the population. Here, the local gene pool is structured as a result of heterogeneity in the accessory gene content that varies among the three dominant lineages (ST5, ST8, ST30) and between animal- and host-derived isolates. These findings suggest that variable host-associated selective pressures imposed on *S. aureus* impacts their genome content, which creates multiple avenues to achieving lineage success. Notably, the three dominant lineages are major clones that have been implicated in a variety of human infections [37]. ST5 and ST8 are also both considered pandemic lineages associated with the global dissemination of methicillin resistance [38]. ST30 is associated with hospital- and community-acquired MRSA [37, 38]. Their ability to cause infections in animals further underscores the importance of how their generalist lifestyle and ability to infect multiple hosts can facilitate the widespread dissemination of clinically relevant adaptive genes. The three lineages vary in their repertoire of AMR and virulence genes. We found differences in the distribution of functional capsule operon among the three lineages, and such differences have been previously reported to be associated with variation in epidemiological patterns and disease presentation [39]. The host range of the *rep* plasmid families and the SCC*mec* types, which carry different suites of cargo genes, also contribute in part to the accessory genome variation between lineages and host sources.

Our results highlight the importance of low-frequency lineages, regardless of the identity of their host, as a major source of genetic variation for the dominant STs and to the overall population. Here, only seven out of 75 STs were frequently detected. The rest consisted of divergent lineages or variants of the more common STs. A broader sampling of animal species will likely yield novel STs that remain to be discovered and characterized. As we have shown, these low-frequency lineages act as major reservoir of accessible DNA, and therefore provides an almost endless supply of new allelic variants of core genes or novel accessory genes that the more dominant lineages can draw from. The most frequently recombined genes that we identified (*clfB*, *aroA*, *sraP*) function in host infection and virulence, whose recombination involved these rare lineages. While acquired DNA is often neutral or nearly neutral in effect [40], some are potentially adaptive and provides a selective benefit to the recipient [41]. Even a slight fitness advantage is sufficient for a lineage to succeed. ST5, ST8, and ST30 are frequent recipients of these recombined DNA and we can postulate that their success lies in part to genetic variants and accessory genes, including *clfB*, *aroA*, and *sraP,* acquired from the rare lineages. Moreover, the rarity of microbial lineages is not a fixed characteristic [42]. Although some rare lineages may exhibit persistently low-abundance distributions and occupation of a narrow niche, some may periodically or occasionally increase in abundance when provided with optimal growth conditions [42]. Regardless if or how often their abundance changes, these rare lineages act as important reservoir of ecological potential. Future work will benefit from the long-term monitoring of how these less common STs vary in their host distribution, prevalence, and genome composition over time.

Our findings are consistent with previous population studies of *S. aureus* that span wide geographical scales and lengthy time periods (*i.e.*, thousands of years), which revealed that horizontally acquired or recombined genes are instrumental in the ability of *S. aureus* to infect multiple vertebrate hosts [13, 14, 16]. What we demonstrate in our work, which spans slightly over ten years of sampling, is that the ability of *S. aureus* to acquire beneficial DNA conferring the capacity for survival in the new host is equally relevant when we also consider short evolutionary time scales. Knowledge about how recombination can facilitate short-term adaptation is key to understanding the underlying causes for the persistence of high-risk clones (as in the case of ST5, ST8, ST30) as well as the success of emerging lineages that can potentially become prevalent. Lineages with novel combinations of recombined genetic variants will help uncover key elements in host–pathogen interactions, which could be targeted for effective therapeutic measures. This knowledge is also relevant to identifying key routes of human-animal transmission and host switches, which are pertinent to mitigating opportunities for disease or antimicrobial resistant strains to emerge at human-animal interfaces. Beyond *S. aureus*, our findings will be relevant to other bacterial species with the capacity to infect and disperse between humans and animals.

A strength of our study is its targeted focus on *S. aureus* sampled from the same geographical region, although sampled at partly overlapping time periods between the human and animal hosts. However, we recognize the limitations of our study. There were three main sources of sampling bias in our dataset. First, our dataset is heavily favored toward human-derived isolates. This was resolved by our analysis of the subsampled dataset consisting of the same number of isolates from human and animals, which recapitulated the recombination results obtained from our entire dataset. Second, we grouped all animal-derived isolates together, even though each animal species represents a distinct ecological niche and presents a unique selective pressure on bacterial pathogens. By grouping together the animal isolates, the signatures of recombination and genomic variation we identified could be specific to human hosts. The animal host species where our bacterial samples came from were also disproportionately represented by domestic animals (cow, dog, cat) and less so from wildlife species. Wildlife is known to harbor *S. aureus*, including MRSA [43]. Third, the isolation site was substantially different for each group. While all human isolates represented bloodstream infections, animal isolates were largely isolated from skin and soft tissue infections and milk. Not only do these biases potentially affect the genome size and makeup of the core and accessory gene content, including the distribution of AMR and virulence genes, but it is possible that certain STs were overrepresented and rare ones were overlooked. We also did not determine recombined genes originating from other species or taxa outside of our dataset, which can also be important sources of genetic variation. We also did not have information about direct contacts between human and animal hosts (*e.g.*, pet owners and their companion animals, farmers and their livestock); hence, we were unable to precisely determine whether the recombination events we have inferred were recent and to what extent they were engendered by these host interactions. Notwithstanding these limitations, our results provide a robust population genomic comparison of *S. aureus* from different vertebrate hosts, which can be used as basis for a more systematic sampling schemes in the future.

## Conclusions

We have shown that the remarkable ability of *S. aureus* as a versatile, multi-host pathogen lies in part on the heterogeneity in the accessory genomes of the dominant lineages (ST5, ST8, ST30), and the frequent recombination with low-frequency lineages that act as an important reservoir of host-associated genetic variants. These findings have direct implication for designing effective measures for targeted mitigation of public and veterinary health threats, including controlling the dissemination of antimicrobial resistant strains across the One Health continuum [44]. Our study highlights the importance of continuous surveillance of *S. aureus* lineages and genetic elements circulating in animal and human hosts, as well as the need for systematic sampling across different ecological host niches. Our work provides an essential framework for future investigations to better understand the ability of *S. aureus* and other bacterial pathogens to infect and adapt to multiple host species.

## Materials and methods

### Genomic dataset

We retrieved a total of 437 published genome sequences of *S. aureus* isolates that we have previously characterized [25, 26]. This dataset consisted of genomes from 323 *S. aureus* isolates from unique pediatric and adult patient blood cultures [25], whereas 114 isolates were obtained through routine diagnostic tests of clinical specimens from animals with confirmed clinical infections [26]. We collected a single isolate per patient or animal. The human-derived isolates were archived from blood cultures submitted to the Department of Pathology and Laboratory Medicine at the DHMC, New Hampshire, USA from December 2010 – August 2018. The animal-derived isolates were obtained from culture swabs from routine clinical specimens submitted to the NHVDL, New Hampshire, USA from September 2017 – March 2020. Samples were received by the NHVDL from multiple veterinary practices from four states (New Hampshire, Maine, Massachusetts, and Vermont) located in the northern New England region in the northeastern part of the country. The dataset consisted of genomes sequenced using Illumina HiSeq platform (San Diego, California). Initial genome characterization included de novo assembly pipeline using Shovill v1.1.0 (https://github.com/tseemann/shovill) and further assembly quality check using QUAST v5.0.2 [45] and CheckM v1.1.3 [46]. The dataset included only genomes with < 200 contigs and an N50 > 40,000 bp. Genomes were annotated using Prokka v1.14.6 [47]. We calculated the genome-wide Average Nucleotide Identity (ANI) for every possible pair of genomes using fastANI v.1.32 [48].

### Determination of the core and accessory genomes

Pangenome analysis was carried out to determine the core and accessory gene content. We used Roary v.3.13.0 [49] to cluster protein sequences using CD-HIT [50], all-against-all BLASTP [51], and Markov clustering [52]. Nucleotide sequences were aligned using MAFFT v.7.477 [53]. We defined core genes as those present in ≥ 99% of the genomes (*i.e.*, 433 genomes), whereas the accessory genes were genes present in ≥ 0% and < 99% of the 437 genomes. Genes found in only a single genome (*i.e.*, unique genes) were filtered from the pan-genome and functionally annotated using eggNOG-mapper v2.1.9 [54, 55]. For every pair of genomes, we calculated the genetic distance based on SNPs of the concatenated alignment of the core genes using snp-dist v0.8.2 (https://github.com/tseemann/snp-dists). We also measured the core SNP distance only among the human isolates and only among the animal isolates based on the core genome of each group. We used the same definition of the core genome described above.

### Phylogenetic and population structure analysis

A core genome phylogeny was built from the core genome alignment of the entire dataset (437 genomes) and the reference genome of *Staphylococcus argenteus* MSHR1132 (NCBI accession number: GCA_000236925.1) as outgroup. From this alignment, we extracted the SNPs using SNP-site v.2.5.1 [56] and built a maximum likelihood phylogenetic tree using the program RAxML v.8.2.12 [57] with a generalized time reversible (GTR) [58] model of nucleotide substitution and Gamma distribution of rate heterogeneity. We used *S. argenteus* as the outgroup to root our tree following the approach in previous studies [59–62]. The outgroup was then removed, and the tree was reconstructed using the core SNP alignment obtained only for the 437 *S. aureus* genomes, with the cluster closest to the outgroup as the most ancestral. We also reconstructed the phylogenies of individual genes (*clfB, aroA* and *sraP*, which were identified as frequently recombining). Using the gene sequence alignment generated from Roary [49], we built maximum likelihood phylogenetic trees using the program RAxML v.8.2.12 [57] with the GTR + Gamma model of nucleotide substitution. Phylogenetic trees were visualized using the online platform Interactive Tree of Life (ItoL) [63].

We used the Bayesian hierarchical clustering algorithm fastBAPS v1.0.6 (fast Bayesian Analysis of Population Structure) [64] to partition the genomes into sequence clusters consisting of genetically similar individuals. While the ST of the isolates have been assigned previously [25, 26], we repeated the typing using mlst v2.19.0 (https://github.com/tseemann/mlst) to incorporate any updates or addition of new STs in the pubMLST database [65] since the last time these genomes were analyzed. The STs are based on sequence variation in seven single-copy housekeeping genes (*arc*, *aroE*, *glpF*, *gmk*, *pta*, *tpi, yqiL*) [27]. Finally, we calculated the genome-wide ANI for all possible pairs of genomes from the three dominant lineages (ST5, ST8 and ST30) using the program FastANI v.1.32[48].

### Principal component analysis

We used PCA to determine the maximum variation in the pan-genome data (*i.e.*, totality of genes in the dataset). Two separate files containing the core and accessory matrices were used as input in the R 4.1.2 [66] package recipes v1.0.1 (https://github.com/tidymodels/recipes). We ran the PCA analysis on genomes partitioned according to host (human versus animal) and according to STs (ST5, ST8 and ST30). The script and the files used for the PCA analysis are public available (https://github.com/stephaniesrsouza/PCA-Core-and-Accessory-S.-aureus.git).

### In silico detection of antimicrobial resistance and virulence genes, SCC*mec*, and plasmids type

Identification of antimicrobial resistance genes and virulence factors was carried out using ABRicate v1.0.1 (https://github.com/tseemann/abricate) by screening the Comprehensive Antibiotic Resistance Database (CARD) [67] and the Virulence Factor Database (VFDB) [68]. We used the minimum thresholds of > 80% for sequence coverage and > 80% sequence identity when we compared our sequences to the sequences in the databases. We determined the presence and types of the mobile element staphylococcal chromosomal cassette *mec* (SCC*mec*) using staphopia-sccmec v1.0.0 [69]. Lastly, we inferred the presence of plasmids by screening the PlasmidFinder database [70] using ABRicate v1.0.1 (https://github.com/tseemann/abricate).

### Estimating homologous recombination

We used two methods to detect homologous recombination events in *S. aureus* genomes.

First, we calculated the relative rates at which polymorphisms are introduced from recombination (rho ρ) and mutation (theta θ) using ClonalFrameML v1.12 [71] with -emsim set at 100 simulations and the true option for -embranch argument. The ρ/θ ratio is a measure of how often recombination events happen relative to mutations [72]. As input to ClonalFrameML, we used the maximum likelihood phylogenetic tree built using RaxML v.8.2.12 [57] with GTR + Gamma based on the full alignment of the concatenated core genes of the entire dataset. Using the same approach, we also calculated the ρ/θ ratio for each of the three dominant lineages (ST5, ST8 and ST30). We also used snp-dist v0.8.2 (https://github.com/tseemann/snp-dists) to calculate the pairwise core SNP distance within each lineage before and after recombination. To calculate the genetic distance after the recombination, we applied the cfml-maskrc script (https://github.com/kwongj/cfml-maskrc) to mask the recombinant regions from the alignment and then calculated the pairwise core SNP distance.

Second, we inferred the presence of recombination events in individual core genes and shared accessory genes of the entire *S. aureus* dataset using fastGEAR [73]. For each sequence alignment, fastGEAR uses a Hidden Markov Model to identify polymorphic sites occurring in a strain and compares them to other polymorphic sites occurring in members of its own lineage as well as strains from other lineages. We parsed the output of fastGEAR in HERO (Highways Enumerated by Recombination Events) (https://github.com/therealcooperpark/hero), a pipeline implemented in Python to visualize donor-recipient strain pairs in recent recombination events identified by fastGEAR. Visualization of recombination events was carried out using Circos v.0.69–8 [74]. To overcome potential biases due to the sample size difference between animal and human isolates, we resampled our human isolates to match the number of animal isolates. A subset of 114 human isolates were randomly selected using the sample function in base v.4.1.2 package (https://www.rdocumentation.org/packages/base/versions/3.6.2) in R 4.1 (66). We then run this smaller dataset of 228 isolates (114 human isolates and 114 animal isolates) in Roary to determine the core and accessory genome as described above. Later, we re-analyzed the recombination events and the donor-recipient pairs using fastGEAR and HERO as described above.

### Statistical analysis

We carried out all statistical analysis using the ggstastsplot v.0.9.4 package in R 4.1. [66]. We used Welch’s t-test to compare the following parameters: the number of unique genes, coding sequences (CDS), accessory genes, recombination events, total genome size, rho/theta values, plasmid diversity, and core SNP distance between animal and human isolates. The same statistical test was also applied to compare the core SNP distance before and after recombinant regions were removed and the plasmid content of ST5, ST8 and ST30. Results were considered significant when *p* < 0.05.

### Supplementary Information

**Additional file 1: Supplementary Table S1.** Accession numbers, associated metadata, ST, BAPS sequence cluster, homologous recombination information, AMR and virulence genes and rep families of the 437 S. aureus genomes in this study. **Supplementary Table S2.** Functional classification of the unique genes (i.e., isolate-specific) detected in human and animal isolates according to eggnog. **Supplementary Table S3.** Genome-wide average nucleotide identity (ANI) values for every pair of S. aureus genomes. **Supplementary Table S4.** List of recombination events predicted by ClonalFrameML indicating the initial position and end position of each recombination event detected in the genomes as well as in the ancestor nodes. **Supplementary Table S5**. Recombination events inferred from the 437 genomes using fastGEAR. The results include information about the donor group, recipient group, start/end position of event, gene name, and a list of recipient genomes. **Supplementary Table S6.** Recombination events inferred from a subset of 228 genomes (114 human and 114 animals) using fastGEAR. The results include information about the donor group, recipient group, start/end position of event, gene name, and a list of recipient genomes.

**Additional file 2: Supplementary Figure S1.** Phylogenetic tree based on core SNP of 437 *S. aureus *genomes. The maximum likelihood tree was built using 1,563 core genes. *Staphylococcus argenteus *strain MSHR1132 (NCBI accession number: GCA_000236925.1) was used as the outgroup. Colored branches represent the 12 sequence clusters inferred by the program BAPS. The two outer stripes represent the host and sequence type (ST) of each genome. Branch scale represents the number of nucleotide substitutions per site. **Supplementary Figure S2.** Genome characteristics of animal- and human-derived *S. aureus *isolates. Comparison of (a) genome size, (b) coding sequences (CDS), (c) pairwise core genome SNP distance, (d) number of accessory genes, and (e) number of unique genes. In panels a, b, d, and f, the red dots represent the mean number. In panel c, the blue dashed line represents the overall mean for all pairwise comparisons. Significance was tested using Welch’s t-test. **Supplementary Figure S3.** Heatmap of average nucleotide identity (ANI) values for every possible pair of genomes between any two of the three dominant STs (a) ST5 and ST8, (b) ST5 and ST30, and (c) ST8 and ST30. ANI values are found in Supplementary Table S3. **Supplementary Figure S4.** Comparison of human- and animal-derived isolates among the three dominant STs (ST5, ST8, and ST30) showing the (a) the number of accessory genes, (b) the number of different *rep *families and (c) distribution of AMR and virulence determinants. Intensity of box shading indicates the proportion of genomes harboring at least one gene conferring resistance to an antimicrobial class (red) and virulence mechanism (blue). In panels a and b dots represent genomes in each category. In the violin plots, the mean value is represented by the red dot, the vertical line in the middle of the violin represents the standard deviation. Details of the distribution of specific genes related to AMR, virulence and plasmids are presented in Supplementary Table S1. Significance was tested using Welch’s t-test. **Supplementary Figure S5.** Donors and recipients in recombination events of core and shared accessory genes for a subset of 228 isolates (114 isolates from animals + 114 isolates from human). For each of the three dominant STs (ST5, ST8, ST30), we subdivided the genomes into whether they came from a human host or animal host. We grouped all low-frequency STs into the Other STs category, which were further subdivided into whether they were human-derived (dark gray outer ring) or animal-derived (light gray outer ring). The length of the outer block is proportional to the number of genomes in which recombination was detected. Each line in the center connects a pair of donor and recipient genomes. For each line, the recipient genome is indicated by the end of the line ending nearer the outside of the plot and the potential donor cluster is indicated by the end of the line nearer the center of the plot. The color of the connecting line is based on the color of the recipient genome. The width of the lines in the center is proportional to the number of recombination events between a pair of genomes from the respective groups. The list of all recombination events is presented in Supplementary Table S5. **Supplementary Figure S6.** Maximum likelihood phylogenies of the three frequently recombined genes calculated using fastGEAR. (a) Gene tree calculated from the *aroA *alignment of 437 *S. aureus *included in this study. (b) Gene tree calculated from the *clfB *alignment of 326 *S. aureus *carrying the respective gene. (c) Gene tree calculated from the *sraP *alignment of 385 *S. aureus *carrying the respective gene. Because the number of genomes differed among the three trees, we used midpoint-rooting. For all trees, the colors out of the branches represent the sequence types (STs), while the colors on the outer ring represent the host from which the isolate was sampled from. Branch scale represents the number of nucleotide substitutions per site.

## Data Availability

The dataset supporting the conclusions of this article is included within the article and its supplementary files. Genome sequence data of *S. aureus* isolates are available NCBI Sequence Read Archive under BioProject accession numbers PRJNA741582 (*S. aureus* from animals) and PRJNA673382 (*S. aureus* from human). BioSample accession numbers for each genome are listed in Supplementary Table 1.

## Abbreviations

MLST: Multi-locus sequence typing
ST: Sequence Typing
MRSA: Methicillin-resistant *S. aureus*
CC: Clonal Complex
SNP: Single Nucleotide Polymorphism
BAPS: Bayesian Analysis of Population Structure
PCA: Principal Component Analysis
CDS: Coding Sequences
AMR: Antimicrobial Resistance
SCCmec: Staphylococcal Cassette Chromosome *mec*
ANI: Average Nucleotide Identity
