# Supplementary material for: Multi-host infection and phylogenetically diverse lineages shape the recombination and gene pool dynamics of Staphylococcus aureus
Source: BMC Microbiol. 2023 Aug 25;23:235. doi: 10.1186/s12866-023-02985-9 (PMC10463932; doi:10.1186/s12866-023-02985-9)
Supplement: Supplementary file 2 — Additional file 2: Supplementary Figure S1. Phylogenetic tree based on core SNP of 437 S. aureus genomes. The maximum likelihood tree was built using 1,563 core genes. Staphylococcus argenteus strain MSHR1132 (NCBI accession number: GCA_000236925.1) was used as the outgroup. Colored branches represent the 12 sequence clusters inferred by the program BAPS. The two outer stripes represent the host and sequence type (ST) of each genome. Branch scale represents the number of nucleotide substitutions per site. Supplementary Figure S2. Genome characteristics of animal- and human-derived S. aureus isolates. Comparison of (a) genome size, (b) coding sequences (CDS), (c) pairwise core genome SNP distance, (d) number of accessory genes, and (e) number of unique genes. In panels a, b, d, and f, the red dots represent the mean number. In panel c, the blue dashed line represents the overall mean for all pairwise comparisons. Significance was tested using Welch’s t-test. Supplementary Figure S3. Heatmap of average nucleotide identity (ANI) values for every possible pair of genomes between any two of the three dominant STs (a) ST5 and ST8, (b) ST5 and ST30, and (c) ST8 and ST30. ANI values are found in Supplementary Table S3. Supplementary Figure S4. Comparison of human- and animal-derived isolates among the three dominant STs (ST5, ST8, and ST30) showing the (a) the number of accessory genes, (b) the number of different rep families and (c) distribution of AMR and virulence determinants. Intensity of box shading indicates the proportion of genomes harboring at least one gene conferring resistance to an antimicrobial class (red) and virulence mechanism (blue). In panels a and b dots represent genomes in each category. In the violin plots, the mean value is represented by the red dot, the vertical line in the middle of the violin represents the standard deviation. Details of the distribution of specific genes related to AMR, virulence and plasmids are presented in Supplementary Tab [file 12866_2023_2985_MOESM2_ESM.pdf]

# Multi-host infection and phylogenetically diverse lineages shape the recombination and gene pool dynamics of *Staphylococcus aureus*

Stephanie S.R. Souza, Joshua T. Smith, Spencer A. Bruce, Robert Gibson, Isabella W. Martin, Cheryl P. Andam

Supplementary Material

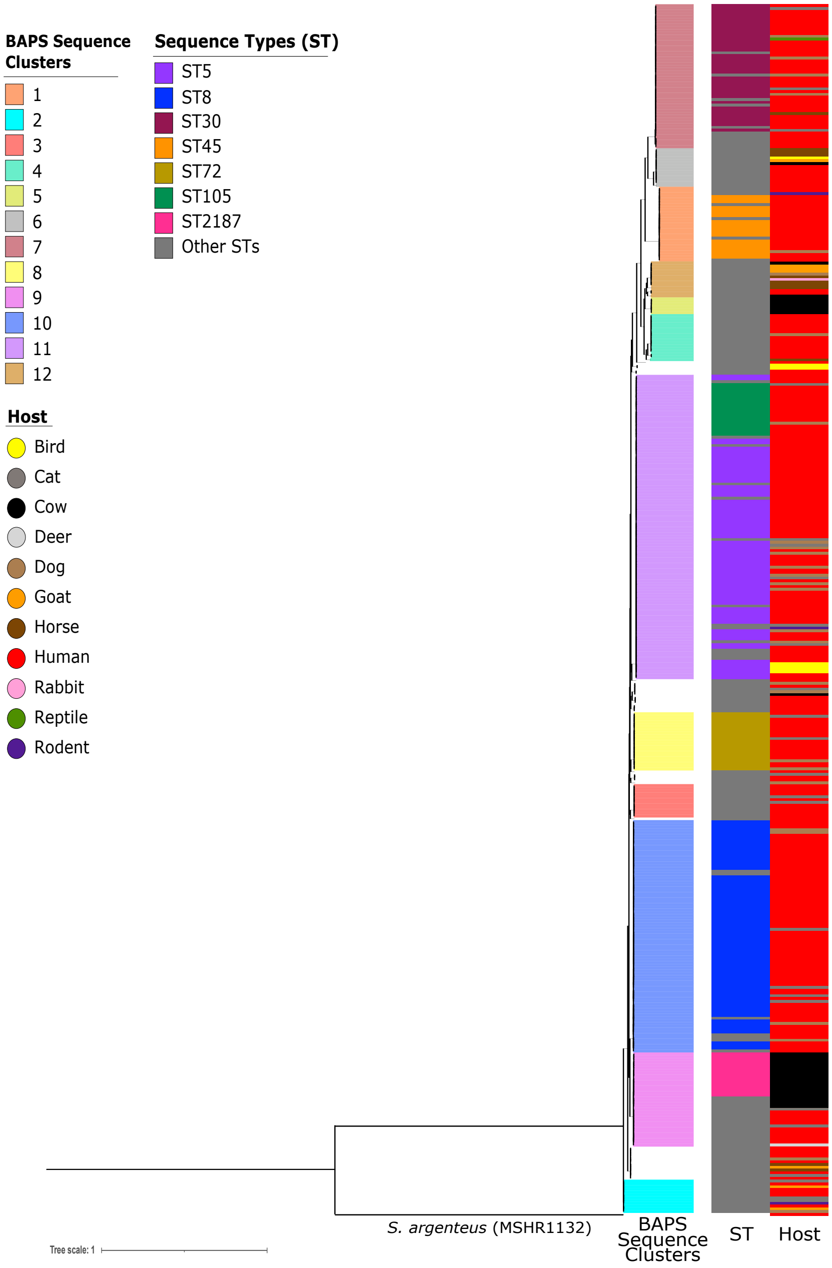


**Supplementary Figure S1.** Phylogenetic tree based on core SNP of 437 *S. aureus* genomes. The maximum likelihood tree was built using 1,563 core genes. *Staphylococcus argenteus* strain MSHR1132 (NCBI accession number: GCA_000236925.1) was used as the outgroup. Colored branches represent the 12 sequence clusters inferred by the program BAPS. The two outer stripes represent the host and sequence type (ST) of each genome. Branch scale represents the number of nucleotide substitutions per site.

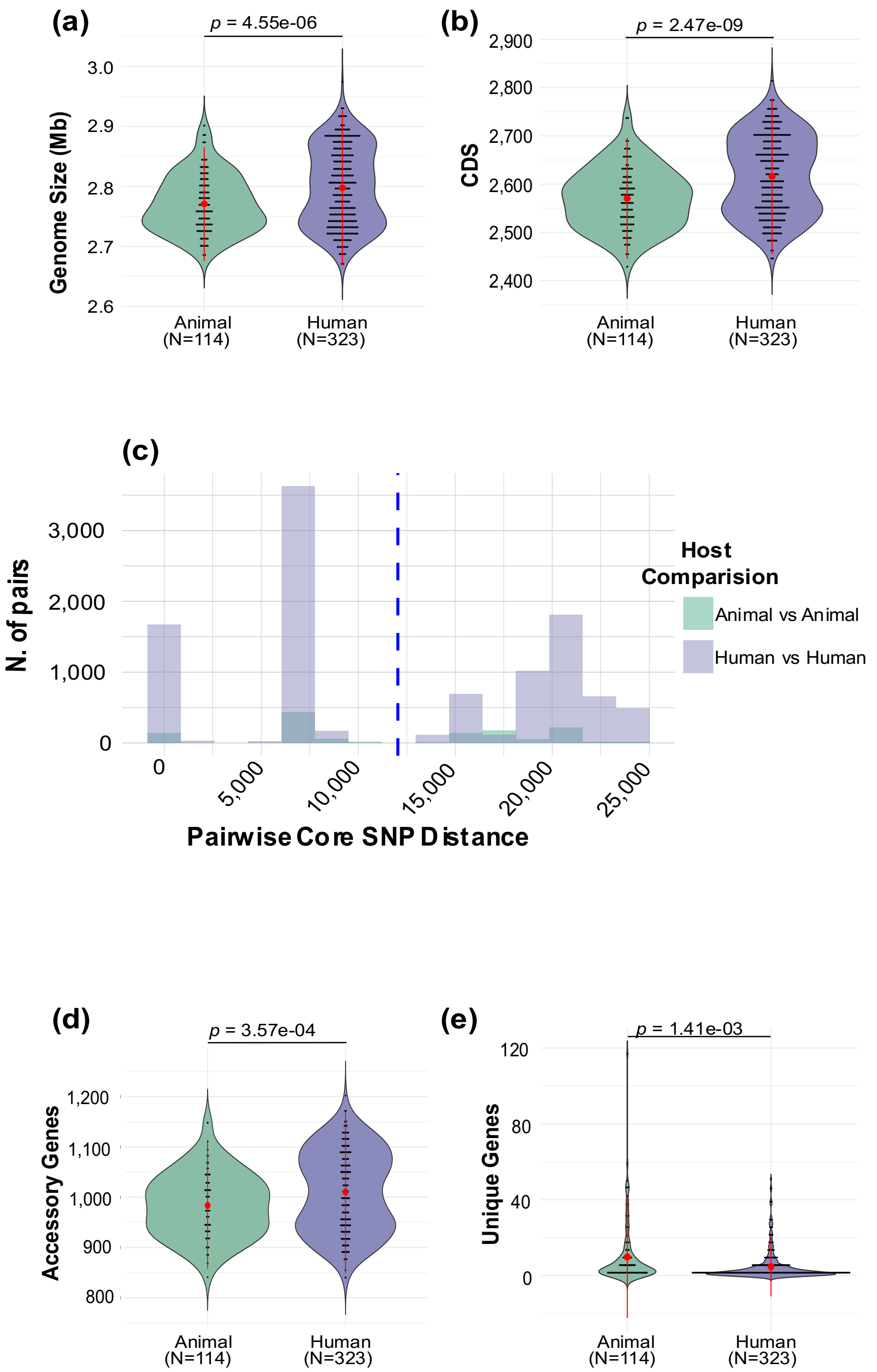


**Supplementary Figure S2.** Genome characteristics of animal- and human-derived *S. aureus* isolates. Comparison of (a) genome size, (b) coding sequences (CDS), (c) pairwise core genome SNP distance, (d) number of accessory genes, and (e) number of unique genes. In panels a, b, d, and f, the red dots represent the mean number. In panel c, the blue dashed line represents the overall mean for all pairwise comparisons. Significance was tested using Welch's t-test.

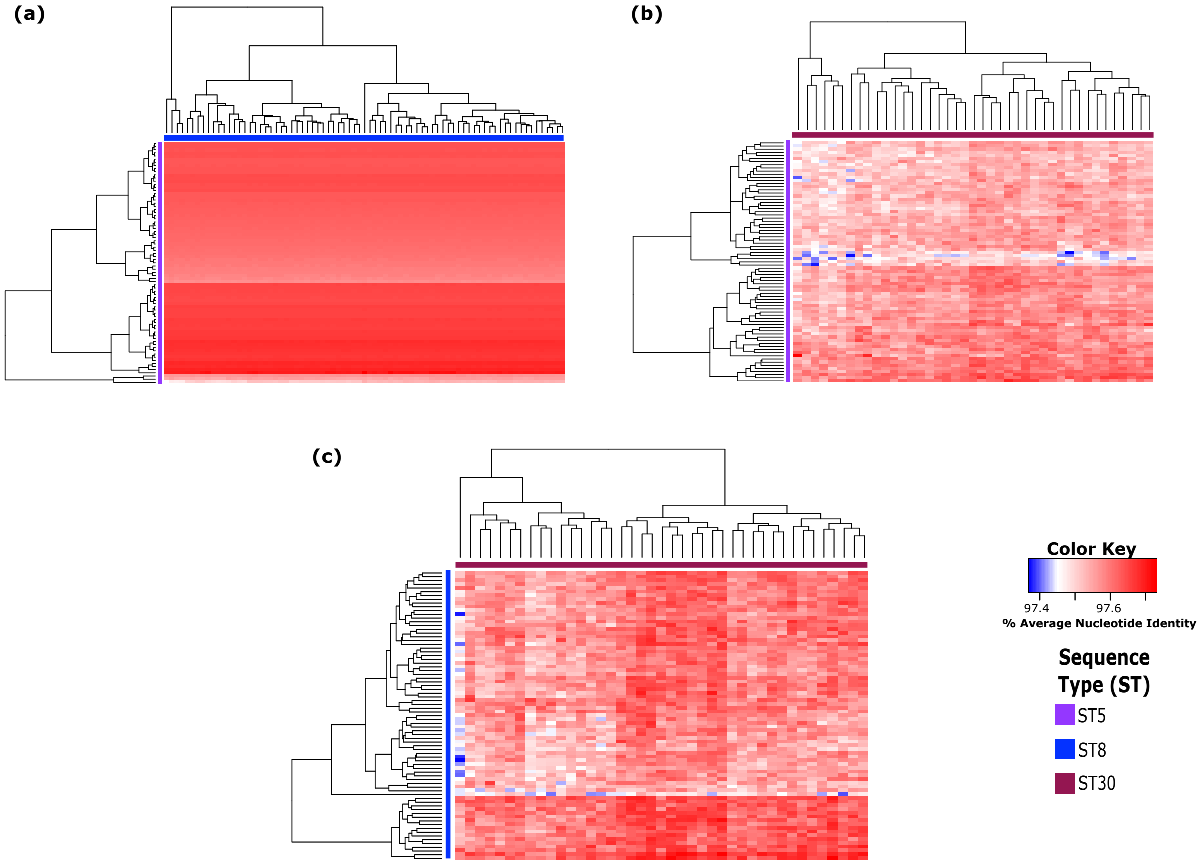


**Supplementary Figure S3.** Heatmap of average nucleotide identity (ANI) values for every possible pair of genomes between any two of the three dominant STs (a) ST5 and ST8, (b) ST5 and ST30, and (c) ST8 and ST30. ANI values are found in Supplementary Table S3.

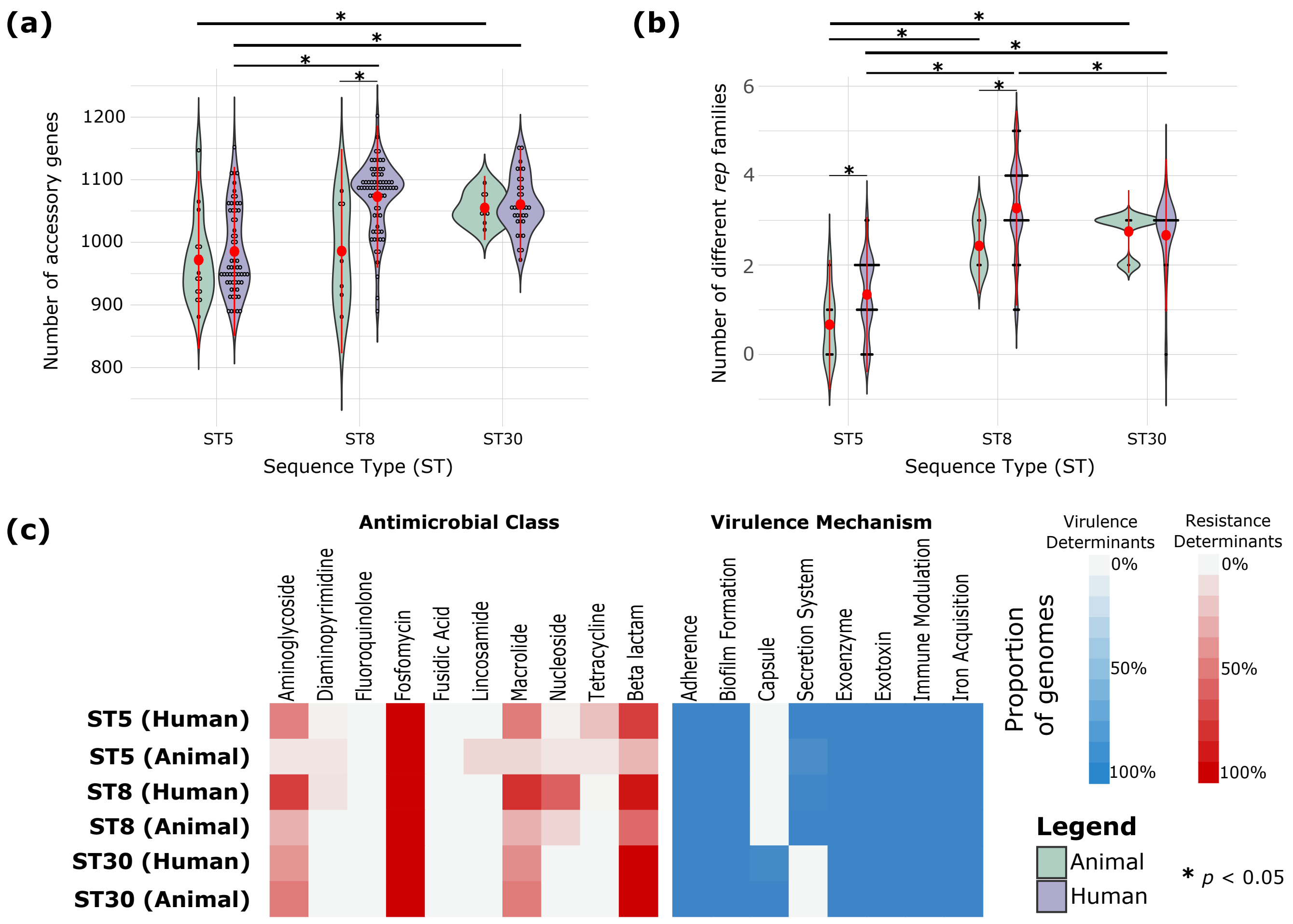


**Supplementary Figure S4.** Comparison of human- and animal-derived isolates among the three dominant STs (ST5, ST8, and ST30) showing the (a) the number of accessory genes, (b) the number of different *rep* families and (c) distribution of AMR and virulence determinants. Intensity of box shading indicates the proportion of genomes harboring at least one gene conferring resistance to an antimicrobial class (red) and virulence mechanism (blue). In panels a and b dots represent genomes in each category. In the violin plots, the mean value is represented by the red dot, the vertical line in the middle of the violin represents the standard deviation. Details of the distribution of specific genes related to AMR, virulence and plasmids are presented in Supplementary Table S1. Significance was tested using Welch's t-test.

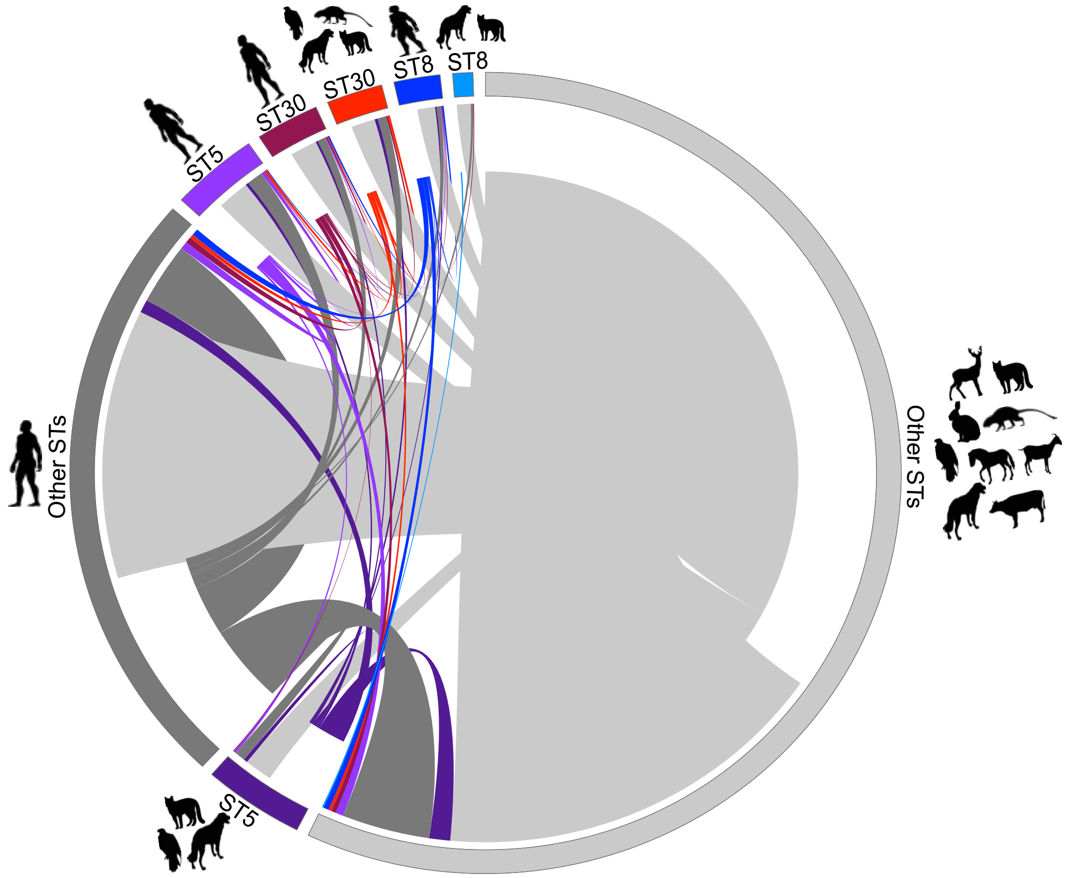


**Supplementary Figure S5.** Donors and recipients in recombination events of core and shared accessory genes for a subset of 228 isolates (114 isolates from animals + 114 isolates from human). For each of the three dominant STs (ST5, ST8, ST30), we subdivided the genomes into whether they came from a human host or animal host. We grouped all low-frequency STs into the Other STs category, which were further subdivided into whether they were human-derived (dark gray outer ring) or animal-derived (light gray outer ring). The length of the outer block is proportional to the number of genomes in which recombination was detected. Each line in the center connects a pair of donor and recipient genomes. For each line, the recipient genome is indicated by the end of the line ending nearer the outside of the plot and the potential donor cluster is indicated by the end of the line nearer the center of the plot. The color of the connecting line is based on the color of the recipient genome. The width of the lines in the center is proportional to the number of recombination events between a pair of genomes from the respective groups. The list of all recombination events is presented in Supplementary Table S5.

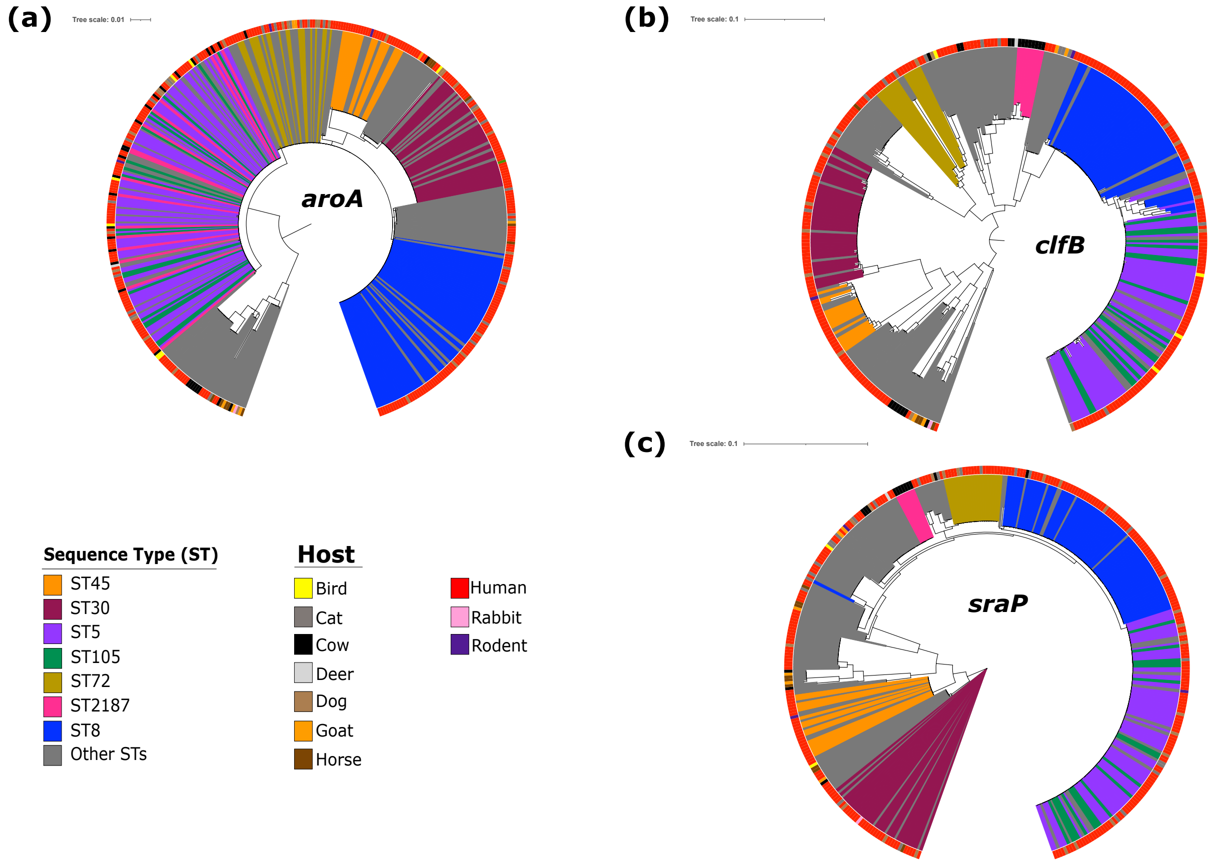


**Supplementary Figure S6.** Maximum likelihood phylogenies of the three frequently recombined genes calculated using fastGEAR. (a) Gene tree calculated from the *aroA* alignment of 437 *S. aureus* included in this study. (b) Gene tree calculated from the *clfB* alignment of 326 *S. aureus* carrying the respective gene. (c) Gene tree calculated from the *sraP* alignment of 385 *S. aureus* carrying the respective gene. Because the number of genomes differed among the three trees, we used midpoint-rooting. For all trees, the colors out of the branches represent the sequence types (STs), while the colors on the outer ring represent the host from which the isolate was sampled from. Branch scale represents the number of nucleotide substitutions per site.
